# Monitoring of hepatitis E virus RNA during treatment for chronic hepatitis E virus infection after renal transplantation

**DOI:** 10.1002/iid3.411

**Published:** 2021-02-08

**Authors:** Patrick Affeldt, Veronica Di Cristanziano, Franziska Grundmann, Maike Wirtz, Rolf Kaiser, Thomas Benzing, Dirk Stippel, Martin Kann, Christine Kurschat

**Affiliations:** ^1^ Department II of Internal Medicine and Center for Molecular Medicine Cologne, Faculty of Medicine, University Hospital Cologne University of Cologne Cologne Germany; ^2^ Faculty of Medicine, University Hospital of Cologne, Institute of Virology University of Cologne Cologne Germany; ^3^ Department of General, Visceral and Cancer Surgery, University Hospital of Cologne University of Cologne Cologne Germany

**Keywords:** chronic HEV infection, ELISPOT, ribavirin, rituximab, serial HEV RNA monitoring, SOT

## Abstract

**Background:**

Recently, chronic hepatitis E virus (HEV) infections gained increasing attention as a possible cause for elevated liver enzymes of unknown origin and liver cirrhosis in solid organ transplant recipients. Reduction of immunosuppressive therapy and/or use of antiviral drug ribavirin have been established as possible treatment strategies.

**Methods:**

The efficacy of dose reduction of mycophenolic acid (MPA) and ribavirin therapy was retrospectively analyzed in eight renal transplant patients of our outpatient clinic who were diagnosed with HEV infection by detection of specific antibodies (immunoglobulin M and immunoglobulin G) and/or positive RNA in blood and stool. In four patients serial HEV viral loads in blood were measured.

**Results:**

Only one patient reached HEV clearance after reduction of immunosuppressive therapy (predominantly reduction of MPA daily dose) alone, whereas six patients were treated with ribavirin after reduction of immunosuppressive therapy due to persistent virus replication. Four of six patients reached HEV clearance after 3 months of ribavirin therapy. HEV clearance was observed after 34–42 days. Two patients, both treated with rituximab within the last 12 months before diagnosis of HEV infection, needed prolonged ribavirin therapy due to persistent viral replication.

**Conclusion:**

Reduction of daily dose of MPA therapy alone in transplant patients with chronic HEV infection may not be sufficient to control viral replication. HEV clearance under ribavirin therapy shows interindividual variability. Therefore, serial viral monitoring may be useful to personalize treatment duration. Rituximab therapy is a risk factor for complicated‐to‐treat chronic HEV infection.

## Graphical abstract

In this article, hepatitis E virus (HEV) infections in renal transplant recipients were examined. Serial measurements of HEV‐RNA are helpful to determine the optimal duration of ribavirin treatment. We identified previous rituximab treatment to be a risk factor for a prolonged treatment course.

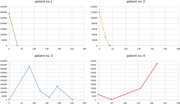

## 1 INTRODUCTION

Elevated liver enzymes as a marker of liver injury in patients without any known preexisting liver disease are a frequent finding in renal transplant recipients.^1^ The etiology of liver injury in those patients is heterogeneous and includes bacterial, fungal, and viral infections as well as noninfectious causes like drug toxicity or malignancies.^1^, ^2^

Recently, hepatitis E virus (HEV) infection gained increasing attention as a possible cause for hepatitis. Within the general population in Germany, HEV specific IgG can be detected in 16.8%.^3^ Among immunocompetent patients, HEV infections usually lead to an acute self‐limiting hepatitis and only few cases of chronic infections have been described.^4^ Therefore, a specific treatment is not necessary in the vast majority of patients. In contrast, chronic courses of HEV (genotype 3) infection with up to 10% of rapid development of liver cirrhosis can occur in immunosuppressed patients ^4^, ^5^, ^6^ having undergone solid organ transplantation. Chronic hepatitis E infection poses a significant risk to patients and graft survival, respectively, and—due to the lack of an approved therapy—a challenge to the counseling physicians.

As a first line treatment approach, the reduction of immunosuppressive therapy has been established. According to the literature, up to 30% of HEV‐infected patients show a spontaneous clearance of HEV after reduction of immunosuppression at a follow up of 6 months.^6^ In the absence of spontaneous clearance, the treatment option of choice is ribavirin,^7^ resulting in HEV clearance after 3 months in up to 78%–100% of patients.^4^, ^7^, ^8^ However, ribavirin therapy carries the risk of adverse effects, such as anemia due to bone marrow toxicity.^7^, ^9^

The appropriate treatment of HEV infection in immunosuppressed patients remains controversial, mostly due to the lack of controlled trials and overall small numbers of cases.^4^

The present case series is focused on immunosuppressed patients which have been tested positive for HEV within 3 years following kidney transplantation. The aim is to share our experience of treatment options regarding mycophenolic acid (MPA) dose reduction as well as ribavirin therapy and possible risks factors for initial treatment failure.

## 2 MATERIALS AND METHODS

All kidney transplant recipients testing positive for HEV (positive immunoglobulin M [IgM] and immunoglobulin G [IgG] and/or positive RNA) in clinical routine diagnostics obtained at the outpatient clinic of Department of Nephrology of the University Hospital of Cologne, Germany, between January 2016 and March 2020 were included in this case series. Demographic and clinical data as well as laboratory markers (e.g., for kidney function and liver injury), HEV specific antibodies (IgM and IgG), and HEV‐RNA load in blood and stool by real‐time PCR (polymerase chain reaction) were summarized. Treatment decisions including the reduction of immunosuppressive therapy or initiation of antiviral therapy with ribavirin are reported. All parameters were examined in clinical routine care for patients and were summarized retrospectively. Patients showing a HEV viremia persisting for more than 3 months were considered as chronically infected.^10^

### 2.1 HEV diagnostics

Specific HEV IgM and IgG were detected using the recomLine HEV IgG/IgM immunoassay (Mikrogen diagnostic), according to the manufacturer's instructions. HEV‐RNA detection in blood and stool was performed using the RealStar HEV RT‐PCR Kit 2.0 (Altona), according to the manufacturer's instructions.

### 2.2 Statistical analyses

Data are reported as medians (interquartile range [IQR]), or frequencies (*n* [% of total]). Thereafter, to determine significant changes in HEV‐RNA blood levels a Wilcoxon matched pairs signed rank test was performed for HEV‐RNA blood levels, lymphocyte, and leucocyte count before and after modification of immunosuppressive therapy. A Spearman correlation was performed for correlation between lymphocyte count an HEV‐RNA blood levels. All statistical testing was two‐sided and a *p* < .05 was considered significant. All analyses were performed using SPSS (Statistical Package for Social Sciences, SPSS Inc., version 24.1) software.

## 3 RESULTS

All eight patients were male and had a history of elevated liver enzymes, which lead to further laboratory diagnostics and diagnosis of chronic HEV infection.

The median age at time of diagnosis was 57.5 years (25–69 years) (Table 1). One patient died 2 weeks after diagnosis of HEV infection and subsequent reduction of immunosuppressive therapy due to liver failure caused by hepatocellular carcinoma and liver cirrhosis of undetermined cause (patient no. 8). In the remaining seven patients, the median time between date of transplantation and diagnosis of HEV infection was 33 months (IQR: 46 months).

**Table 1:** Patient baseline characteristic

	*n* = 8
Male sex, no%	100
Age, years	58.5 (25–69)
Immunosuppressive regime	
Triple with tacrolimus	8
Triple with cyclosporine A	0
Dual with tacrolimus	0
Dual with cyclosporine A	0
mTor Inhibitor, MPA, Steroid	0
Tacrolimus median plasma levels (µg/dl)	5.9
Induction therapy	
Basiliximab (d0, d4)	8
Lymphocyte depleting agents	0
Liver enzyme elevation at time of first positive HEV‐PCR measurement	
ALT (ref.: male, <41 U/l	8
AST (ref.: male, <50 U/l)	8
ɣGT (ref.: male 8–61 U/l)	8
Time between transplantation and first HEV‐RNA measurement, months (IQR)	33 (46)

Abbreviations: ALT, alanine aminotransferase; AST, aspartate aminotransferase; ɣGT, gamma‐glutamyl transpeptidase; HEV, hepatitis E virus; IQR, interquartile range; MPA, mycophenolic acid.

The source and etiology of HEV infection in six of eight patients remained unclear. Two patients (no. 3 and 7) were huntsmen and had a history of eating self‐hunted venison. All patients except patient no. 3, showed positive IgM and IgG response to HEV. Patient no. 3 had received rituximab 5 months before an AB0‐incompatible living donor kidney transplantation and did not show serologic immune reactivity against HEV despite positive PCR results. In contrast, patient no. 4 had developed a serologic immune reactivity 12 months after having received rituximab due to recurrence of his underlying renal disease (pauci‐immune glomerulonephritis).

At the time of diagnosis, all patients were treated with a triple immunosuppressive therapy, including tacrolimus, MPA and prednisone (Table 1). All patients received basiliximab as induction therapy on Days 0 and 4. In addition patient no. 3 was treated with immunoadsorption and rituximab before transplantation. Before reduction of immunosuppressive therapy, median HEV‐RNA plasma copy numbers were 1.47E+06 IU/ml (IQR: 1.16E+09 IU/ml) (Table 2). In two patients, initial HEV viremia was not quantified as the diagnosis was initially established in an external outpatient clinic (no. 5 and 6).

**Table 2:** HEV‐RNA blood levels, leucocyte, and lymphocyte count before and 3 months after reduction of immunosuppressive therapy

	Before IS reduction		After IS reduction		
	HEV‐RNA IU/ml	Leucocytes (Lymphocytes)	HEV‐RNA IU/ml	Leucocytes (lymphocytes)	MPA dose reduction %
Patient 1	2.06E+06	13.00 × 10E9/L (1,00 × 10E9/L)	2.86E+05	13.00 × 10E9/L (0.91 × 10E9/L)	25
Patient 2	1.84E+06	7.16 × 10E9/L (2.36 × 10E9/L)	6.22E+05	6.1 × 10E9/L (1.82 × 10E9/L)	50
Patient 3	9.00E+03	3.28 × 10E9/L (0.98 × 10E9/L	2.49E+03	2.93 × 10E9/L (0.74 × 10E9/L)	50
Patient 4	7.16E+05	5.10 × 10E9/L (0.86 × 10E9/L)	2.57E+05	5.1 × 10E9/L (0.49 × 10E9/L)	25
Patient 5	§	§	§	§	100
Patient 6	§	12.43 × 10E9/L (1.27 × 10E9/L)	§	11.24 × 10E9/L (0.95 × 10E9/L)	50
Patient 7	1.09E+06	7.07 × 10E9/L (1.2 × 10E9/L)	0	11.09 × 10E9/l (4.57 × 10E9/L)	50
Patient 8	4.62E+09	1.09 × 10E9/L (0.05 × 10E9/L)	‡	‡	100
Median (IQR)	1.47E+06 (1.16E+09)		2.57E+05 (4.53E+05)		
Sig. (2‐tailed)"			0.028	0.75 (0.345)	

*Note*: ^‡^Patient no. 8 died before finishing the 3‐month interval; ^§^HEV‐RNA levels, leucocyte, and lymphocyte counts were missing because of external diagnostic and later on treatment initiation in our clinic.

Abbreviations: IQR, interquartile range; IS, immunosuppressive therapy; MPA, mycophenolic acid; Sig., two tailed significance of spearman correlation.

The median plasma level of tacrolimus at time of diagnosis was 5.9 (3.4–11.2) µg/L (Table 1). Upon diagnosis, the daily dose of tacrolimus was initially reduced in patient no. 2 because of modification of target trough level. Patient no. 5 received a dose reduction of tacrolimus because of elevated serum levels (11.2 mg/dl). In all HEV‐infected patients, as a first step of HEV treatment, MPA therapy was reduced (minimum reduction to 25% of the initial dose) at time of diagnosis. In one patient (no. 5), MPA was initially lowered to 50% of the initial dose for 3 months and then discontinued for the following 3 months.

After reduction of immunosuppressive therapy, viral load in blood and stool was monitored for at least 3 months. After 3 months, HEV‐RNA was cleared in one patient (no. 7) only. Three to five months after reduction of immunosuppressive therapy, the median HEV‐RNA plasma level decreased from 1.47E+06 to 2.27E+05 IU/ml. There was no patient with an increase in HEV‐RNA plasma level after this therapy adjustment. Decline in HEV‐RNA plasma levels before and after reduction of immunosuppressive therapy was significant (*p* = .028) (Table 2). Total count of lymphocytes and leucocytes before and after reduction of immunosuppressive therapy was measured (Table 2). Neither total lymphocytes nor leucocytes count changes were significant (leucocytes *p* = .198, lymphocytes *p* = .068). Total lymphocyte count before and after reduction of immunosuppressive therapy was not significant correlated to HEV‐RNA plasma levels before and after reduction of immunosuppressive therapy (*p* = .313).

Only patient no. 7 reached HEV clearance after reduction of immunosuppressive therapy alone, patient no. 8 died within 2 weeks from liver cancer not related to HEV infection. Since a high viral load was still present even after reduction of immunosuppressive therapy in the remaining six patients, they were started on ribavirin therapy with a dose adjusted to their renal function. The median ribavirin dose was 300 mg per day (200–800 mg) (Table 3), 800 mg of ribavirin were administered in only one patient (no. 2).

**Table 3:** Ribavirin therapy

	Ribavirin initial dose (mg/dl)	Change of ribavirin daily dose due to anaemia (%)	Serum creatinine at start of ribavirin treatment (mg/dl)	eGFR at start of ribavirin treatment (ml/min)	Time to first negative blood HEV‐PCR (days)	HEV clearance after 3 months of ribavirin therapy
Patient 1	300	nr	1.83	44	34	yes
Patient 2	800	75%	1.47	77	42	yes
Patient 3	250	nr	1.53	50	254	no
Patient 4	200	nr	3.13	21	†	no
Patient 5	300	nr	1.63	45	nm	yes
Patient 6	400	nr	1.26	67	nm	yes
median	300		1.58	47.5		

*Note*: Patient no. 3 (CMV coinfection) reached HEV clearance after 9 months of ribavirin therapy. ^†^Patient no. 4 is still on therapy after 7 months of treatment. ^†^No HEV clearence after 7 months of ribavirin therapy.

Abbreviations: CMV, cytomegalovirus; GFR, glomerular filtration rate (CKD‐EPI) in ml/min; HEV, hepatitis E virus; nm, no measurement of HEV‐PCR within the first 3 months; nr, no reduction of ribavirin daily dose.

Several adverse drug reactions were observed. The only patient on 800 mg ribavirin starting dose required a dose reduction during the course of the treatment because of anemia due to ribavirin‐induced bone marrow toxicity. After gradual dose reduction to 200 mg, anemia resolved to normal hemoglobin levels (Table 3). Patient no. 3 also suffering from concomitant cytomegalovirus (CMV) infection developed pancytopenia likely caused by added effects of CMV infection as well as MPA and valganciclovir medication. MPA therapy was discontinued, ribavirin was halted for 2 weeks. After resolution of pancytopenia, ribavirin therapy was restarted, and a stable blood count was observed. In the remaining four patients, no severe side effects requiring dose reduction or pausing of ribavirin therapy were observed (Table 3). Because of the CMV‐coinfection, the specific cellular immune response to CMV was measured by T‐SPOT. CMV (Oxford Immunotec) in patient no. 3. Two months before diagnosis of HEV infection, the ELISPOT (Enzyme Linked Immuno Spot) has shown a good functionality of T cell activity.

**Figure 1:**
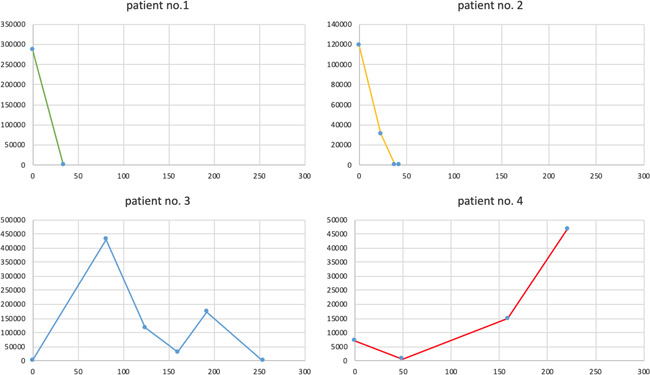
HEV‐RNA in blood under ribavirin therapy. Measured HEV‐RNA IU/ml in blood for every patient. day: day of ribavirin therapy. HEV, hepatitis E virus

In four patients (no. 1, 2, 3, and 4), HEV‐RNA plasma levels were repeatedly measured during the course of ribavirin therapy (Table 4 and Figure 1). In those patients (no. 1 and 2) with successful controlled virus replication after 12 weeks, HEV clearance was achieved after 34 and 42 days, respectively. After 12 weeks of ribavirin therapy, complete HEV clearance in blood and stool was observed in four of six patients (patients no. 1, 2, 5, and 6). In these four patients, liver enzyme values returned to normal levels within 6 months after completion of ribavirin therapy, indicating stable HEV clearance. Patient no. 3 with concomitant CMV infection did not reach HEV clearance after 3 months of ribavirin therapy. Therefore, duration of ribavirin therapy was prolonged to 9 months, till HEV clearance was achieved. In the other patient (no. 4) who did not reach HEV clearance after 3 months of therapy, HEV plasma levels increased from 7.15 E+03 IU/ml (start of ribavirin therapy) to 4.68 E+04 IU/ml after 9 months of ribavirin therapy. Patient no. 4 has not reach HEV clearance yet and is still under ribavirin therapy at the time of writing of this manuscript (August 2020). Both patients not having achieved HEV clearance after 3 months of ribavirin therapy had a history of rituximab therapy within the last 12 months due to AB0‐incompatible living donor kidney transplantation (no. 3) or recurrence of the underlying renal disease (pauci‐immune glomerulonephritis) (no. 4).

**Table 4:** HEV‐RNA in blood under ribavirin therapy

Patient no. 1	Day	HEV‐RNA IU/ml	Patient no. 3	Day	HEV‐RNA IU/ml
	0	286,000		0	2490
	34	0		81	430,000
	90	0		124	118,000
				160	30,700
				192	174,000
				254	0
Patient no. 2	Day	HEV‐RNA IU/ml	Patient no. 4	Day	HEV‐RNA IU/ml
	0	119,000		0	7150
	23	31,200		49	682
	38	21		159	15,000
	42	0		221	46,800

*Note*: Measured HEV‐RNA IU/ml in blood for every patient, day: day of ribavirin therapy.

Abbreviation: HEV, hepatitis E virus.

## 4 DISCUSSION

In this case series, we describe our experience in treatment of chronic HEV infection in kidney transplant recipients visiting the nephrology outpatient clinic. Spontaneous HEV clearance after reduction of immunosuppressive therapy was achieved in one patient only. The majority of patients were thus treated with ribavirin therapy adjusted for kidney function. This treatment approach was able to control infection with only moderate side effects. In only one patient dose reduction was needed because of ribavirin induced anemia.^11^ After dose reduction of ribavirin therapy, anemia resolved to normal Hb levels and the patient gained HEV clearance after 3 months of therapy. In general, ribavirin induced anemia can be treated with epoetin substitution and thus increase tolerability of ribavirin therapy.^12^

Overall, our findings agree with previous evidences concerning gender (mainly male) and source of infection (mainly unknown). All eight patients in our center were of male gender, which is in line with previous findings, as male sex seems to be a risk factor for HEV infection for so far unknown reasons.^2^ In two of the patients, HEV infection was associated with eating venison meat. Similar ways of infection have been described before.^4^, ^5^, ^13^

In one patient, HEV diagnosis was delayed because of missing serologic immune response due to rituximab therapy and immunosuppression in preparation of an AB0‐incompatible living donor kidney transplantation. Such missing serologic response in HEV infections has already been described.^3^, ^14^ This case highlights the necessity to include PCR testing in addition to serological tests for the diagnosis of HEV infection in patients under immunosuppression presenting with unclear elevation of liver enzymes.^4^, ^6^, ^10^

Previous studies described HEV clearance in up to 30% of patients following reduction of immunosuppression.^6^ Kamar et al.^15^ proposed a reduction of tacrolimus trough levels rather than modification of MPA daily dose. According to our internal clinical standard for virus infections, we primary reduced MPA daily dose to get control over viral replication. In this retrospective case series, reduction of MPA daily dose did not lead to spontaneous HEV clearance in most patients, although a significant reduction of HEV viremia was observed. Neither total leukocyte nor total lymphocyte count has changed significantly. In other immunosuppressed cohorts, like allogeneic hematopoietic stem cell transplant recipients, reduction of immunosuppressive therapy was associated with increase of mortality.^16^

In four of our patients, HEV‐RNA blood levels were repeatedly measured during ribavirin therapy. HEV clearance was obtained in the three patients after 34, 42, and 254 days (including 2 weeks of therapy interruption in patient no. 3), respectively (Figure 1 and Table 4). One patient did not reach HEV clearance at the time of writing this manuscript. In the literature there is no clear recommendation for optimal therapy duration due to the heterogeneity of time to archiving HEV clearance.^15^ Overall, 4 out of 6 (67%) patients treated with ribavirin for 3 months obtained HEV clearance. This finding is in line with previous observations, showing more than 80% of viral clearance after 3 months of antiviral treatment with ribavirin.^2^, ^8^, ^9^

Previous studies already identified risk factors for treatment failure of chronic HEV infection. Immunosuppressive therapy with tacrolimus instead of cyclosporine A^6^ and a low lymphocyte count at therapy initiation^7^ have been shown to be associated with a decreased response to therapy. All patients that are described in this case series were on medication with tacrolimus. In our case series 4 out of 6 patients archived HEV clearance after 12 weeks of ribavirin therapy. Those patients with initial treatment failure after 12 weeks of therapy both received rituximab because of AB0‐incompatible living donor kidney transplantation or recurrence of underlying renal disease. There are several case reports discussing the possible impact of rituximab concerning treatment failure of ribavirin therapy for other immunosuppressed patients.^17^, ^18^ Concerning renal transplant recipients, only one case series in the field discussed the risk for difficult to treat HEV infection after rituximab therapy.^19^

## 5 CONCLUSION

In the present investigation on renal transplant recipients affected from chronic HEV infection, a reduction of MPA therapy alone for 3 months was not sufficient to control viral replication.

Dose reduction of MPA appears to result in less viral clearance from reduction of the immunosuppressive therapy alone than reported elsewhere.^15^ Controlled trials are warranted, and tacrolimus trough level reduction may be more effective.

Today, most kidney transplanted patients receive tacrolimus medication.^20^ Taking into account that tacrolimus therapy seems to be a risk factor for treatment failure,^6^ early ribavirin therapy should be evaluated in every patient. In addition reduction of immunosuppressive therapy in other immunosuppressed patients was associated with an increase of mortality.^16^

Concerning optimal duration of ribavirin therapy, controlled trials are still warranted.^15^ As we could show, time to HEV clearance seems to be variable within a large range. In our case series, a minimum of 41 days between first occurred negative PCR for HEV‐RNA in blood and end of ribavirin therapy (patient no. 2) was sufficient to lead to stable HEV clearance. Therefore, we suggest to repeatedly measure HEV‐RNA in stool and blood during ribavirin therapy to define an individual duration of treatment. Controlled trails for optimal duration of ribavirin therapy after negative HEV‐RNA PCR in blood are missing.

In our case series, all patients with initial treatment failure of ribavirin therapy received rituximab within the last year. Therefore, previous rituximab therapy should be noted as a possible risk factor for complicate‐to‐treat HEV infections even if in the presence of an apparently functioning cellular immune response.

## CONFLICT OF INTERESTS

The authors declare that there are no conflict of interests.

## AUTHOR CONTRIBUTIONS

Patrick Affeldt, Veronica Di Cristanziano, Martin Kann, Maike Wirtz, and Christine Kurschat performed the research and collected the data. Patrick Affeldt, Veronica Di Cristanziano, Martin Kann, and Christine Kurschat designed the research study. Patrick Affeldt and Veronica Di Cristanziano analyzed the data. Patrick Affeldt, Veronica Di Cristanziano, Franziska Grundmann, Rolf Kaiser, Thomas Benzing, Martin Kann, and Christine Kurschat wrote the paper.

## ETHICS STATEMENT

Data were collected as part of routine clinical diagnostics. The ethics committee agreed on analyzing data within this project (EK 20‐1313).

## Data Availability

The data that support the findings of this study are available from the corresponding author upon reasonable request.
